# Effects of sex, age, and body mass index on serum bicarbonate

**DOI:** 10.3389/frsle.2023.1195823

**Published:** 2023-07-20

**Authors:** Daisy Duan, Jamie Perin, Adam Osman, Francis Sgambati, Lenise J. Kim, Luu V. Pham, Vsevolod Y. Polotsky, Jonathan C. Jun

**Affiliations:** 1 Division of Endocrinology, Diabetes, and Metabolism, Department of Medicine, Johns Hopkins University School of Medicine, Baltimore, MD, United States; 2 Department of International Health, Johns Hopkins Bloomberg School of Public Health, Baltimore, MD, United States; 3 Division of Pulmonary and Critical Care, Department of Medicine, Johns Hopkins University School of Medicine, Baltimore, MD, United States; 4 Center for Interdisciplinary Sleep Research and Education, Johns Hopkins University School of Medicine, Baltimore, MD, United States; 5 Departments of Anesthesiology and Critical Care Medicine and Pharmacology and Physiology, George Washington University School of Medicine and Health Sciences, Washington, DC, United States

**Keywords:** obesity, sex, BMI, bicarbonate, obesity hypoventilation syndrome, sleep apnea

## Abstract

**Rationale:**

Obesity hypoventilation syndrome (OHS) is often underdiagnosed, with significant morbidity and mortality. Bicarbonate, as a surrogate of arterial carbon dioxide, has been proposed as a screening tool for OHS. Understanding the predictors of serum bicarbonate could provide insights into risk factors for OHS. We hypothesized that the bicarbonate levels would increase with an increase in body mass index (BMI), since the prevalence of OHS increases with obesity.

**Methods:**

We used the TriNetX Research Network, an electronic health record database with de-identified clinical data from participating healthcare organizations across the United States, to identify 93,320 adults without pulmonary or advanced renal diseases who had serum bicarbonate and BMI measurements within 6 months of each other between 2017 and 2022. We used linear regression analysis to examine the associations between bicarbonate and BMI, age, and their interactions for the entire cohort and stratified by sex. We also applied a non-linear machine learning algorithm (XGBoost) to examine the relative importance of age, BMI, sex, race/ethnicity, and obstructive sleep apnea (OSA) status on bicarbonate.

**Results:**

This cohort population was 56% women and 72% white and 80% non-Hispanic individuals, with an average (*SD*) age of 49.4 (17.9) years and a BMI of 29.1 (6.1) kg/m^2^. The mean bicarbonate was 24.8 (2.8) mmol/L, with higher levels in men (mean 25.2 mmol/L) than in women (mean 24.4 mmol/L). We found a small negative association between bicarbonate and BMI, with an expected change of −0.03 mmol/L in bicarbonate for each 1 kg/m^2^ increase in BMI (*p* < 0.001), in the entire cohort and both sexes. We found sex differences in the bicarbonate trajectory with age, with women exhibiting lower bicarbonate values than men until age 50, after which the bicarbonate levels were modestly higher. The non-linear machine learning algorithm similarly revealed that age and sex played larger roles in determining bicarbonate levels than the BMI or OSA status.

**Conclusion:**

Contrary to our hypothesis, BMI is not associated with elevated bicarbonate levels, and age modifies the impact of sex on bicarbonate.

## Introduction

Obesity hypoventilation syndrome (OHS) is defined as awake alveolar hypoventilation (PaCO_2_ > 45 mmHg) in patients with obesity [body mass index (BMI) ≥ 30 kg/m^2^] without an alternative cause. The pathophysiology of OHS is not completely understood but is hypothesized to be due to a combination of restrictive lung mechanics, reduced sensitivity to carbon dioxide (CO_2_), and increased metabolic CO_2_ production (Mokhlesi, 2010). A formal diagnosis of OHS requires an arterial blood gas test showing elevated CO_2_. However, blood gases are difficult to collect and are infrequently obtained in the outpatient setting, contributing to an underdiagnosis of OHS.

CO_2_ accumulation leads to respiratory acidosis, which is buffered by the renal reabsorption of bicarbonate $\left({\text{HCO}}_{3}^{-}\right)$. Thus, in the absence of renal failure or another acid–base disorder, elevated bicarbonate can provide indirect evidence of elevated arterial CO_2_. Based on this physiology, elevated bicarbonate (defined as ≥27 mmol/L) has been proposed as a screening tool for OHS in patients with obesity presenting to sleep clinics (Macavei et al., 2013; Bingol et al., 2015; Manuel et al., 2015) since more than 90% of patients with OHS also have obstructive sleep apnea (OSA). In fact, bicarbonate elevation may precede the development of daytime hypercapnia (Manuel et al., 2015; Herkenrath et al., 2021).

Since the prevalence of OHS increases with obesity, we hypothesized that serum bicarbonate increases linearly with BMI. In addition, some investigators reported a relatively higher prevalence of OHS in postmenopausal women (BaHammam and Almeneessier, 2019). This finding might be attributable to a loss of the respiratory effects of estrogen and progesterone (Saaresranta and Polo, 2002; Preston et al., 2009). Indeed, data from healthy blood donors (McPherson et al., 1978) showed that bicarbonate levels were lower in women until menopause than in men. However, this sex interaction with age has not been examined either in a larger dataset or in the context of different degrees of obesity. Serum bicarbonate levels could be leveraged to understand the demographic determinants of hypoventilation and, by extrapolation, the determinants of OHS.

In this study, we aimed to examine cross-sectional associations between demographic factors such as age, race, ethnicity, and sex; BMI; and serum bicarbonate in a large patient cohort identified from TriNetX, a U.S. electronic health record (EHR) database. Using this database, we screened for adults with contemporaneous BMI and serum bicarbonate values and tested our hypotheses that the bicarbonate levels (a) increase with an increase in BMI and (b) are higher in women than in men after the typical onset of menopause (i.e., 50 years of age) (Khaw, 1992). We also examined the subgroup of this cohort with OSA since this population may be enriched for OHS and therefore exhibit stronger BMI–bicarbonate associations.

## Methods

This is a multicenter, retrospective cross-sectional study using data obtained through TriNetX (Cambridge, MA, USA). TriNetX is a federated health research network that provides de-identified EHR data from participating healthcare organizations (HCOs) across the United States. Details regarding the data source and quality checks have been described in a prior study (Krishnan et al., 2023). TriNetX’s cloud-based feature allows real-time access to de-identified clinical data and analytical tools, and thus, aggregate datasets generated from data queries involving specific parameters vary based on the date of the query. Patients whose data are provided to the TriNetX database are individuals who have undergone routine medical care at the participating HCOs during the prespecified period. The presented data were queried on 24 February 2022, when the TriNetX Research Network was composed of 58 HCOs and contained at least one visit from 83.5 million patients. We queried the TriNetX database to retrospectively identify patients aged 18 to 90 years with a bicarbonate measurement and a BMI measurement within 6 months of each other from 1 January 2017 to 24 February 2022, while excluding patients with pulmonary fibrosis or interstitial lung disease; chronic kidney disease on dialysis, renal tubular acidosis, estimated glomerular filtration rate (eGFR) < 30 mL/min/1.73 m^2^; fludrocortisone, acetazolamide, or sodium bicarbonate on their medication list; diabetic ketoacidosis; any form of shock; or chronic obstructive pulmonary disease. De-identified individual-level data from the EHRs were then abstracted by the Core for Clinical Research Data Acquisition TriNetX team at the Johns Hopkins Institute for Clinical and Translational Research. OSA was identified using these International Classification of Diseases (ICD)-9 and –10 diagnostic codes: ICD-9 code 327.23 Obstructive sleep apnea (adult, pediatric) and ICD-10 code G47.33 Obstructive sleep apnea (adult, pediatric) (Maas et al., 2021; Renno et al., 2021). All forms of congestive heart failure (CHF) were identified using the following validated ICD-9 and ICD-10 diagnostic codes: 428.0 (congestive heart failure, unspecified), 428.1 (left heart failure), 428.2 (systolic heart failure), 428.3 (diastolic heart failure), 428.4 (combined systolic and diastolic heart failure), 428.9 (heart failure, unspecified), I50.1 (left ventricular failure, unspecified), I50.2 (systolic CHF), I50.3 (diastolic CHF), I50.4 (combined systolic and diastolic CHF), I50.8 (other heart failure), and I50.9 (heart failure, unspecified) (Bates et al., 2023). The study was approved by the Johns Hopkins Institutional Review Board (Demographic Predictors of Bicarbonate Level, IRB00313060).

### Statistical methods

We examined demographic characteristics, including age in years, sex, race, and ethnicity, as well as bicarbonate levels and BMI, for the cross-sectional analysis. We examined the linear trend in bicarbonate as a function of BMI and age using linear regression. We also probed for interactions between age and BMI by constructing categories of both age and BMI and examining whether the effect of BMI on bicarbonate was consistent across age categories and, similarly, whether the effect of age on bicarbonate was consistent across BMI categories. Upon the initial visual inspection of the relationship between age (as a continuous variable) and bicarbonate levels, we noted different linear trends based on approximate age groups. We thus created age categories based on these age groups to examine the interactions between age categories and BMI on bicarbonate levels. All regressions were performed for the entire cohort and stratified by sex. Additionally, we performed a subgroup analysis on those with OSA. As CHF could induce hyperventilation (Lorenzi-Filho et al., 1936; Tang et al., 2023) and increased BMI is associated with an increased risk for heart failure (Kenchaiah et al., 2002), we examined the relationship between serum bicarbonate and BMI after excluding patients with CHF diagnoses as a sensitivity analysis.

Since the associations between serum bicarbonate, BMI, and age may be non-linear, we also applied a non-linear machine learning algorithm using eXtreme Gradient Boosting (XGBoost). This method makes relatively few assumptions compared to parametric methods and has been shown to be fast computationally and robust to overfitting (Chen and Guestrin, 2016). We included age, BMI, sex, race, ethnicity, and OSA status as predictors in this algorithm. Because of the small number of candidate predictors, we did not include any preselection criteria for inclusion. To examine the performance of this algorithm-based model, we randomly selected 50% of our cohort and estimated *R*-squared among the predicted and observed serum bicarbonate for the remaining (holdout) 50%. We examined the estimated associations of BMI and age on bicarbonate levels with accumulated local effects, which describe how features (i.e., selected predictors) influence the prediction of a machine learning model on average (Apley and Zhu, 2016). Using an extreme gradient-boosting algorithm, we analyzed the relative importance of each predictor to bicarbonate levels using fractional gain. Similar to our parametric analysis, we estimated the gradient-boosted model for the overall cohort and stratified it by sex. We performed a sensitivity analysis in which the CHF status was included as an additional predictor in the algorithm. All analysis was conducted in R version 4.1.2. The R package “XGBoost” was used to estimate the extreme gradient-boosting algorithm, and the package ALEplot was used to represent the algorithm results graphically.

## Results

**FIGURE 1:**
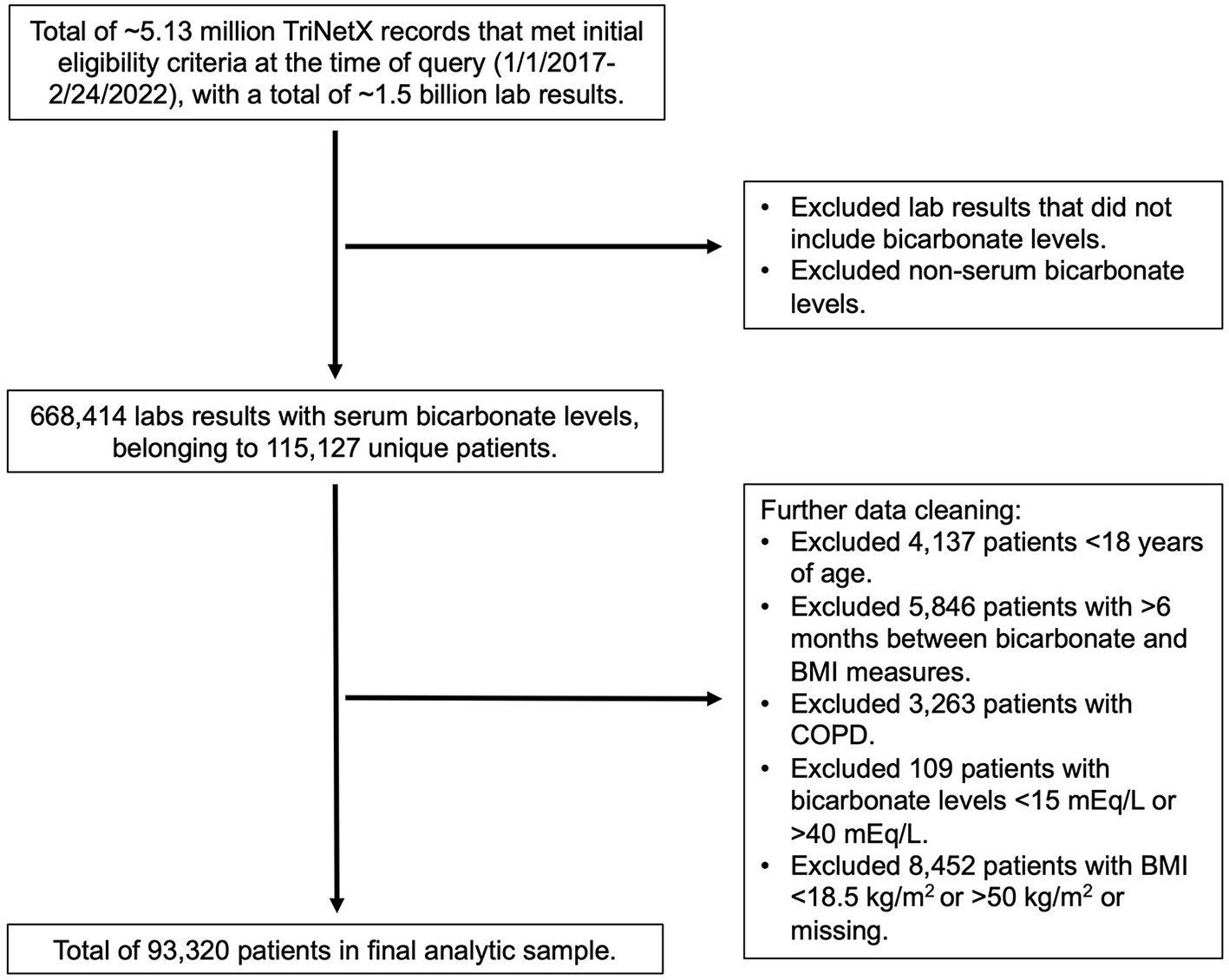
Flow diagram of included and excluded patients in the analytic sample.

The TriNetX query returned ~5.13 million records that met initial eligibility criteria at the time of query (February 24, 2022). From these data, we identified 115,127 unique patients and limited the data to the most recent concomitant ${\text{HCO}}_{3}^{-}$ and BMI measures available. In addition to the exclusion criteria described in the Methods section, we further excluded patients with extreme values, including bicarbonate levels of <15 mmol/L or >40 mmol/L and BMI of <18.5 kg/m^2^ or >50 kg/m^2^, or missing values for BMI measurements. A total of 93,320 patients were included in the final analytic sample for cross-sectional analysis (Figure 1). From this subset, we searched for OSA and CHF using the ICD-9 and ICD-10 diagnostic codes described in the Methods section.

Patient characteristics are described in Table 1. This patient population had an average (*SD*) age of 49.4 (17.9) years and an average (*SD*) BMI of 29.1 (6.1) kg/m^2^. This cohort population was predominantly women (56%), white (72%), and non-Hispanic (80%). The average bicarbonate level in the patient sample was 24.8 (2.8) mmol/L. Serum bicarbonate concentration was greater in men (25.2 mmol/L) than in women (24.4 mmol/L). Approximately 8% of the patients had a diagnosis of OSA, which was more prevalent in men (11%) than in women (6%).

**FIGURE 2:**
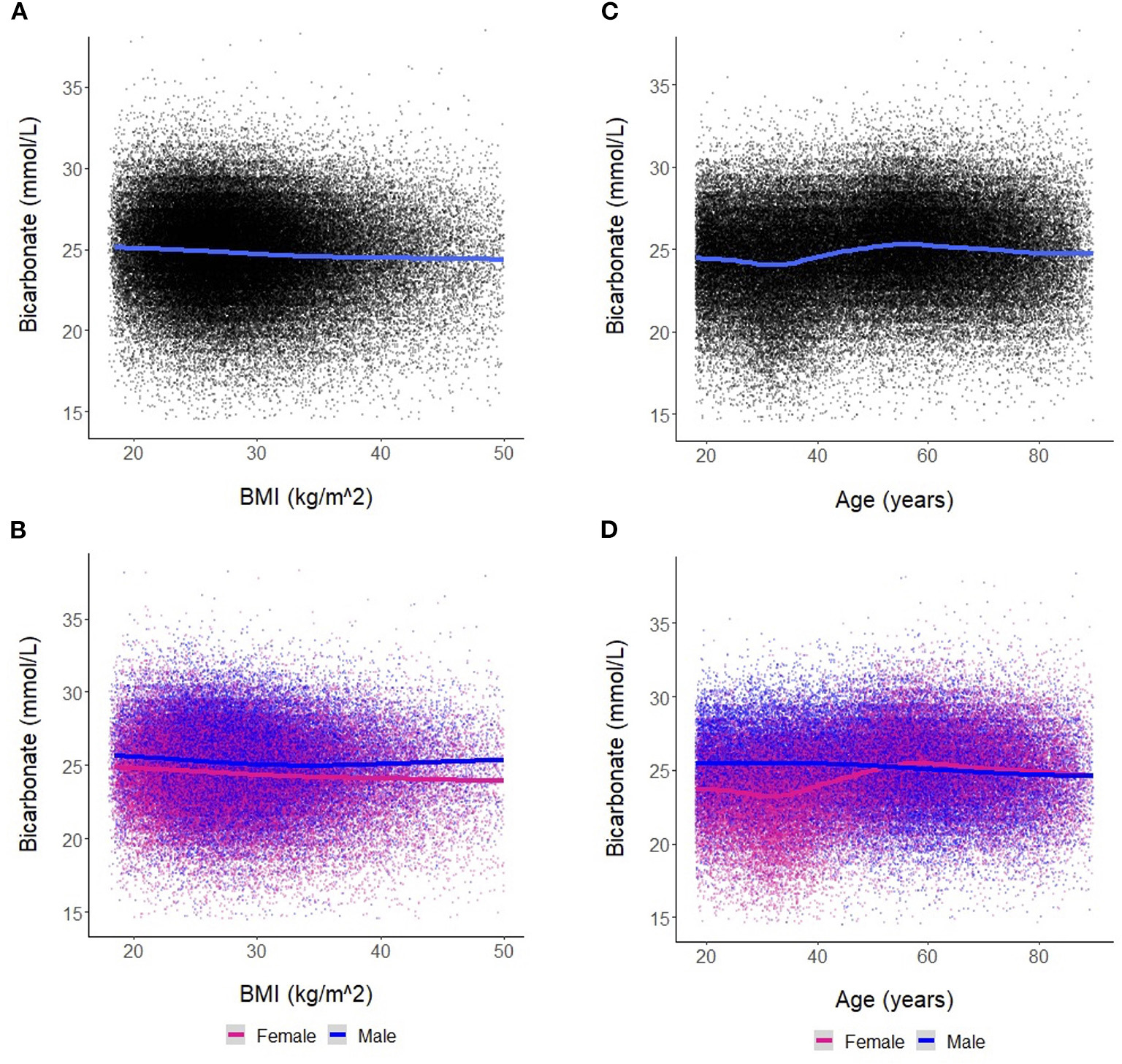
**(A)** Association between bicarbonate levels and body mass index (BMI) for the entire cohort. Each dot represents an individual patient measure (with 0.5 jitter applied to improve visualization of overlapping data), and the line represents a smoothed average of bicarbonate levels as a function of BMI. **(B)** Association between bicarbonate levels and BMI stratified by sex. **(C)** Association between bicarbonate levels and age for the entire cohort. Each dot represents an individual patient measure (with 0.5 jitter applied to improve visualization of overlapping data), and the line represents a smoothed average of bicarbonate levels as a function of age. **(D)** Association between bicarbonate levels and age stratified by sex.

We found a negative linear relationship between serum bicarbonate and BMI (Figure 2A), interpretable as a reduction in serum bicarbonate of −0.030 mmol/L (95% CI [−0.033, −0.027], *p* < 0.001) for each 1 kg/m^2^ increase in BMI. This relationship was similar in men and women (Figure 2B). Among women, the estimated change in bicarbonate was −0.034 mmol/L (95% CI [−0.038, −0.031], *p* < 0.001). Among men, the estimated change in bicarbonate was −0.022 mmol/L (95% CI [−0.026, −0.018], *p* < 0.001). However, men and women differed regarding the trajectory of bicarbonate with age (Figures 2C, D). In men, bicarbonate decreased linearly with age; in women, bicarbonate values were lower than that of men until ~50 years of age. From ages 35–50, bicarbonate levels increased steadily in women while remaining stable in men. After age 50, the average bicarbonate for women exceeded that of men (25.2 mmol/L vs. 25.0 mmol/L, respectively, *p* < 0.001).

**FIGURE 3:**
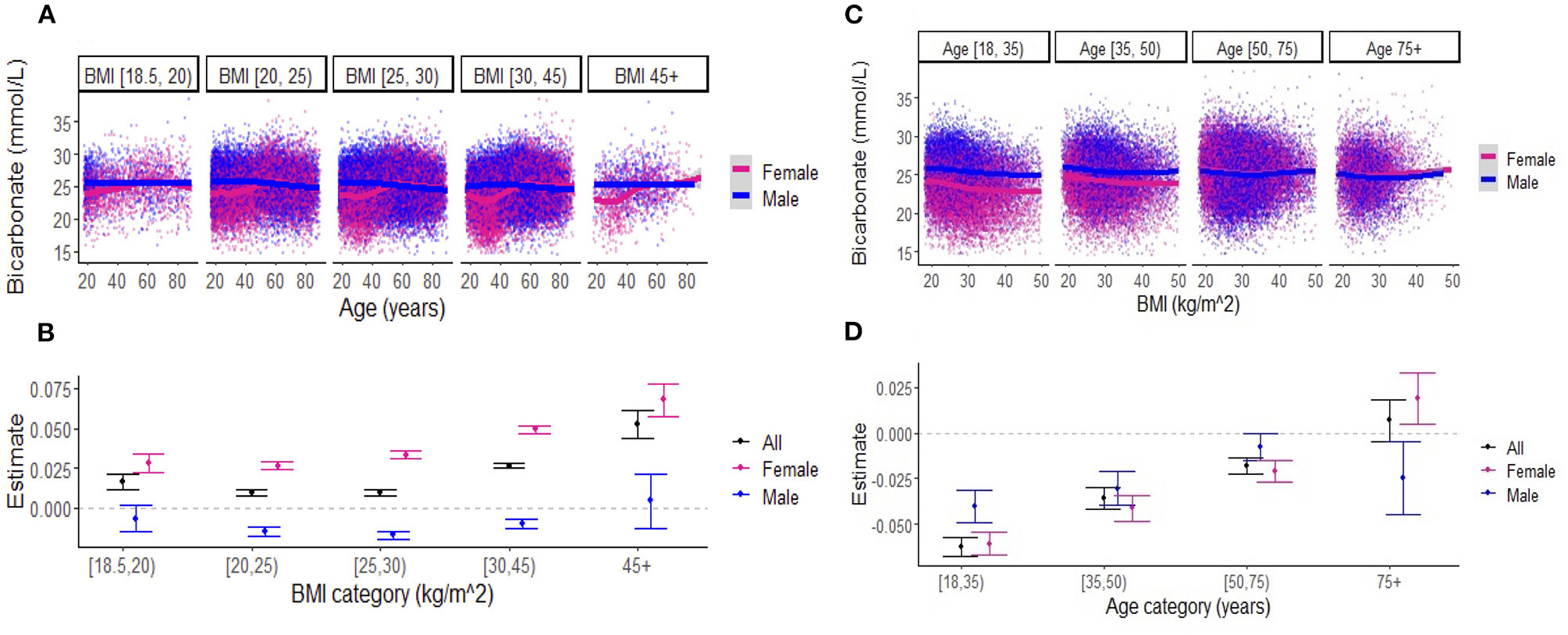
**(A)** Association between serum bicarbonate and age across body mass index (BMI) categories, stratified by sex. Each dot represents an individual patient measure (with 0.5 jitter applied to improve visualization of overlapping data), and the line represents a smoothed average of bicarbonate levels as a function of age, within each BMI category. **(B)** Estimates for the eect of age on serum bicarbonate (outcome) from the linear regression models that include BMI categories (indicator variables), age, and the interactions between BMI categories and age. The reference BMI category was (20–25 kg/m^2^). The numerical values of estimates are summarized in Supplementary Table S1. **(C)** Association between serum bicarbonate and BMI across age categories, stratified by sex. Each dot represents an individual patient measure (with 0.5 jitter applied to improve visualization of overlapping data), and the line represents a smoothed average of bicarbonate levels as a function of BMI, within each age category. **(D)** Estimates for the eect of BMI on serum bicarbonate (outcome) from the linear regression models that include age categories (indicator variables), BMI, and the interactions between age categories and BMI. The reference age category was (18–35 years). Numerical values for estimates are summarized in Supplementary Table S2. Note: [ indicates inclusive; ( indicates exclusive.

When examining the effects of age on serum bicarbonate across BMI categories, there was a difference in the association between bicarbonate levels and age in women vs. men until approximately 50 years of age across all BMI categories (Figure 3A). Linear regression revealed that, in the overall cohort and among women, bicarbonate levels increased with age, and this effect was more pronounced with increasing BMI (Figure 3B; Supplementary Table S1). Interestingly, among men, there was a small negative relationship between bicarbonate and age regardless of BMI category. Similarly, the effects of BMI on bicarbonate levels varied by age group (Figures 3C, D; Supplementary Table S2). In general, there was a negative association between bicarbonate levels and BMI, but this effect diminished with age, until approximately 75 years, when there was a positive association between bicarbonate levels and BMI among women while the inverse relationship between bicarbonate levels and BMI persisted among men. Among those aged between 18 and 75 years, the negative association between BMI and bicarbonate levels was less pronounced among women compared to men.

In the OSA analysis, we noted significant differences in patient characteristics between those with OSA and those without OSA (Supplementary Table S3). Those with OSA had a higher mean bicarbonate level, had a higher mean BMI, were older, and were more likely to be men. Subgroup analysis in the OSA cohort showed similar findings to the overall cohort in the negative linear relationship between bicarbonate levels and BMI, interpretable as a reduction in serum bicarbonate of 0.034 mmol/L [95% CI (−0.042, −0.026), *p* < 0.001] for each 1 kg/m^2^ increase in BMI (Supplementary Figure S1). Note that the linear trends of BMI on bicarbonate levels in men and women were similar and overlapped on the lower end of the BMI spectrum (i.e., BMI < 30 kg/m^2^) but diverged with obesity (Supplementary Figure S1B). This is in contrast to the findings observed in the overall cohort (Figure 2), which demonstrates lower bicarbonate levels in women compared to men throughout the entire BMI spectrum (Figure 2B). In the OSA subgroup, the interactive relationships between age, sex, and BMI on bicarbonate levels were similar to the findings observed in the overall cohort (Supplementary Figure S2). Similarly, the bicarbonate levels were lower in women than men until approximately 50 years of age across all BMI categories (Supplementary Figure S1).

**FIGURE 4:**
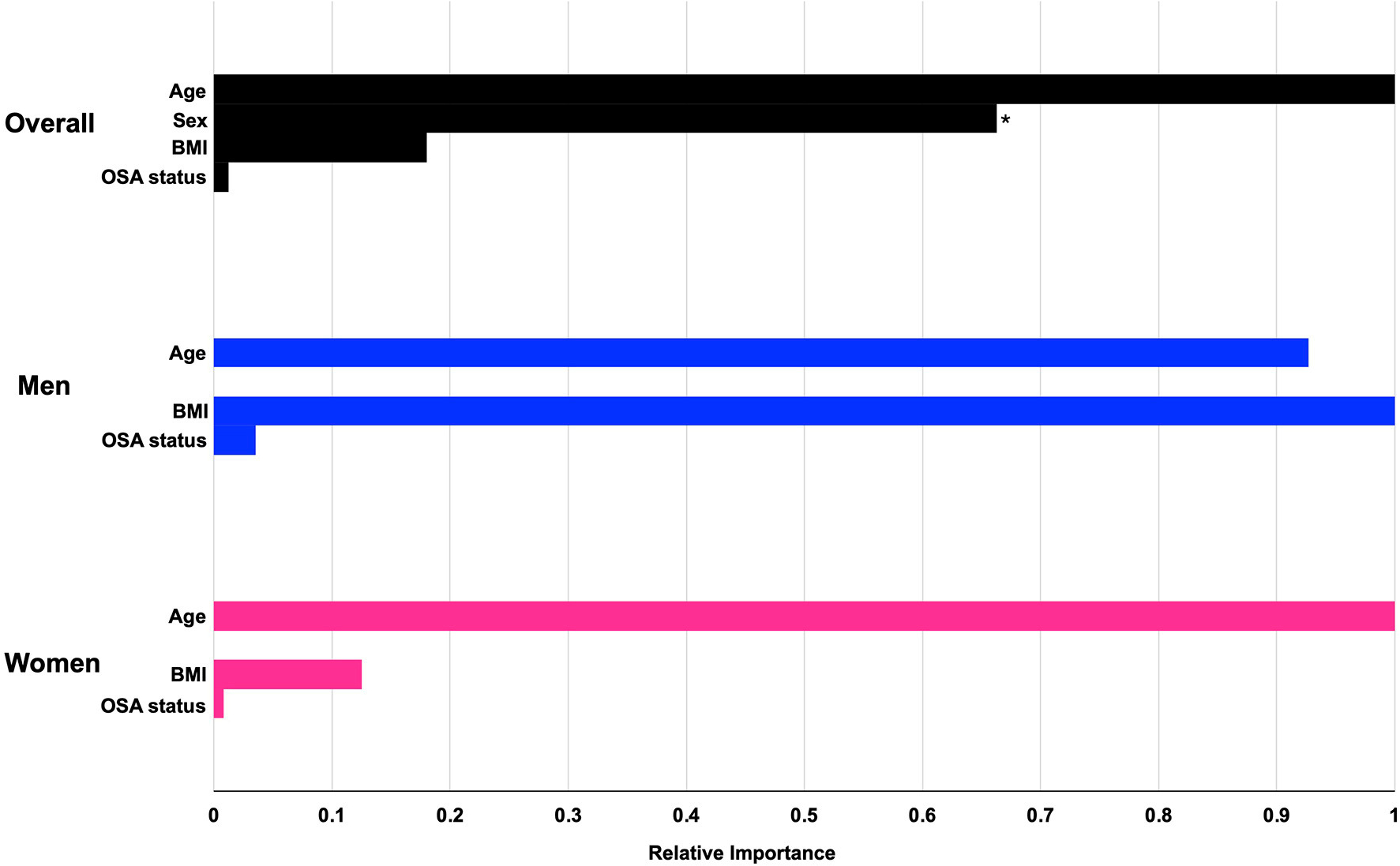
The relative importance of preselected predictors for serum bicarbonate levels from gradient-boosted models. Gradient-boosted model elucidates the relative importance of each predictor on serum bicarbonate levels. Numerical values for fractional gain and relative importance are found in Table 2. *The predictor for sex refers to male sex. OSA, obstructive sleep apnea.

The machine learning algorithm revealed that overall, demographic features, BMI, and OSA status were not strongly predictive of bicarbonate levels, with an estimated *R*-squared of 0.085, 0.026, and 0.095 for the entire cohort, men, and women, respectively. As shown in Figure 4 and Table 2, the most important predictor for the whole cohort, as well as for men and for women separately, was age. The second-most important predictor was sex. Model prediction was not significantly affected by the addition of race and ethnicity to the algorithm, with an estimated *R*-squared of 0.078 for the overall cohort, 0.014 for men, and 0.095 for women and without substantial changes to the fractional gain of the rest of the predictors (data not shown). The accumulated local effects for the gradient-boosted algorithm for age and BMI are shown in Supplementary Figure S3, and the accumulated local effects for age and BMI for the OSA subgroup are shown in Supplementary Figure S4. Estimated changes in bicarbonate levels with age or BMI followed similar patterns as observed in our linear regression analysis. Bicarbonate levels tended to increase with age in women, while they remained constant across all age groups in men. For the overall cohort and both men and women, there was a trend for bicarbonate levels to decrease with an increase in BMI. Changes in bicarbonate levels were difficult to estimate at the extremes of BMI due to a paucity of data. In summary, these observations were consistent with those derived from linear regression analysis.

Approximately 7% of the overall cohort had a diagnosis of CHF (Table 1). Patient characteristics stratified by CHF status are presented in Supplementary Table S4, which showed that those with CHF were much older and were more likely to be men. Univariate linear regression analysis on the association between CHF status and bicarbonate levels showed that bicarbonate levels were lower for those with CHF by 0.40 mmol/L compared to those without CHF [95% CI (−0.43, −0.37), *p* < 0.001]. For our sensitivity analysis, the negative linear association between BMI and serum bicarbonate, excluding those with CHF, was similar to that of the overall cohort (β coefficient −0.033 mmol/L vs. −0.030 mmol/L, respectively). The relationship between age and serum bicarbonate also remained similar after excluding CHF (Supplementary Figure S5). Additionally, including the CHF status in the machine learning algorithm did not change the overall performance of the models in predicting bicarbonate levels, with an estimated *R*-squared of 0.090, 0.030, and 0.098 for the entire cohort, men, and women, respectively. The relative importance of the predictors showed that age remained the most important predictors and sex remained the second most important (data not shown). In conclusion, age and sex are the strongest determinants of bicarbonate levels, in comparison to BMI or EHR-based diagnoses of CHF or OSA.

## Discussion

To our knowledge, this is one of the largest cohort studies examining associations between demographic factors, BMI, and serum bicarbonate. In approximately 93,000 patients from a large U.S. electronic health record network, we found a small negative association between bicarbonate levels and BMI. Women had lower bicarbonate levels than men across the entire BMI spectrum. Notably, women had lower bicarbonate levels than men until 50 years of age, after which women’s bicarbonate levels were modestly higher. Bicarbonate levels were higher among patients with OSA but followed similar trends regarding the effects of age, BMI, and sex. Furthermore, an unbiased machine learning modeling method confirmed that age and sex were more important contributors to bicarbonate levels than BMI or OSA status.

Our findings suggest that obesity, as defined by BMI alone, may be insufficient to cause hypoventilation, assuming that BMI and serum bicarbonate are reasonable metrics/surrogates of obesity and PaCO_2_, respectively. Although this finding refuted our hypothesis, it is in agreement with several prior studies that examined the association of obesity with gas exchange. Several other studies in patients with severe obesity (BMI ≥ 40 kg/m^2^) reported PaCO_2_ in the lower end of the normal range (Zavorsky and Hoffman, 2008). Even in a study of adults with obesity in which PaCO_2_ positively correlated with BMI, 95% of patients had normal PaCO_2_ levels (Gabrielsen et al., 2011). Many people with morbid obesity adopted a rapid respiratory rate to compensate for reduced tidal volume and respiratory system compliance (Littleton, 2011), thereby maintaining alveolar ventilation. A “second hit”, such as defects in leptin signaling (Amorim et al., 2022), might be necessary to cause OHS. It is also possible that BMI does not capture relevant anthropometrics that promote hypoventilation, as metrics of central obesity may be more relevant for OHS risk stratification. For instance, the waist-to-hip ratio (but not BMI) correlated with the PaCO_2_ levels in a study of adults with an average BMI of 49 kg/m^2^ (Zavorsky et al., 2007). As our results demonstrated that serum bicarbonate levels are impacted more by factors such as age, sex, and other unmeasured comorbidities than by BMI, it is also possible that serum bicarbonate is not a reliable surrogate for ventilation.

The inverse association of BMI with serum bicarbonate may be due to the mild tachypnea of obesity described earlier, in the absence of other factors predisposing to OHS. Another factor to consider is the impact of renal function on bicarbonate levels. Obesity may increase the risk of renal impairment, which, in turn, can induce metabolic acidosis. In this study, we excluded patients with stage IV or V chronic kidney disease (eGFR < 30 mL/min/1.73 m^2^), but we still cannot exclude the effects of mild renal dysfunction. More recently, a large-scale retrospective cohort study of more than 96,000 adults visiting outpatient clinics in a single health system found that higher BMIs were associated with both incident low serum bicarbonate levels (defined as ≤23 mmol/L) and progressively lower serum bicarbonate levels over a median follow-up period of 4.4 years (Lambert and Abramowitz, 2021). This finding persisted after excluding those with eGFR of < 60 mL/min/1.73 m^2^ or diabetes. Mechanisms underlying obesity-induced renal dysfunction are postulated to be related to glomerular hyperfiltration, adipokine-mediated direct nephrotoxicity, and ectopic fat deposition in the kidneys (De Vries et al., 2014; Kovesdy et al., 2017). In addition, foods rich in protein can generate precursors that induce mild metabolic acidosis while being a marker of an obesogenic diet (Farhangi et al., 2019). Regardless of the mechanisms involved, our findings suggest that BMI alone may not be a reliable predictor of the bicarbonate level in the general population.

We found that sex modified the relationship between serum bicarbonate and age, similar to earlier reports. McPherson et al. (1978) showed that, in 1,000 healthy blood donors aged 18 to 65 from London, United Kingdom, plasma bicarbonate did not vary with age in men, while the levels were approximately 2 mmol/L (7%) lower until menopause in women, after which the levels converged with men. Interestingly, when stratified by the menopausal status and oral contraceptive use, premenopausal women on oral contraceptives had lower plasma bicarbonate levels compared to premenopausal women not on oral contraceptives, implicating the role of sex hormones. Similarly, Hodgkinson (1982) analyzed data from 436 premenopausal and 117 postmenopausal women and found a significant increase in bicarbonate levels after menopause. Cohort studies from the National Health and Nutrition Examination Survey 1999–2004 (Amodu and Abramowitz, 2013) and the Multi-Ethnic Study of Atherosclerosis (Driver et al., 2014) found that serum bicarbonate levels increased with age, but significant sex differences were not examined (Amodu and Abramowitz, 2013) or detected (Driver et al., 2014).

During menopause, which typically occurs at 50 years of age, progesterone and estrogen levels decline. Prior to menopause, higher progesterone levels in women may stimulate hyperventilation relative to men (Hodgkinson, 1984). Estradiol increases the number of progesterone receptors and may potentiate progesterone’s respiratory stimulant effects (Leavitt and Blaha, 1972; LoMauro and Aliverti, 2015). These hormonal effects are accentuated during the luteal phase of the menstrual cycle (Slatkovska et al., 2006) or during pregnancy when progesterone levels are at their highest (Behan and Wenninger, 2008). The rise in bicarbonate levels in women starting at ~50 years of age may reflect a gradual loss of progesterone and estrogen that accompanies menopause. Small clinical studies have examined the effect of exogenous progesterone in OHS, resulting in improved oxygenation and PaCO_2_ (Sutton et al., 1975), including a study in which intramuscular progesterone was administered in addition to dietary weight loss (Lyons and Huang, 1968). In some cohorts, postmenopausal women accounted for either the majority of OHS cases or presented with more advanced OHS. In a study of 1,973 patients (mean age ~62 years) referred to the sleep clinic for the evaluation of OSA, the prevalence of OHS among women and men was 15.6% and 4.5%, respectively, despite no differences in BMI (BaHammam and Almeneessier, 2019). Notably, the prevalence of OHS among premenopausal women (5.3%) was similar compared with men (4.5%) in the cohort. A Swedish registry of all OHS patients on long-term mechanical ventilation (mean age ~64 years) reported similar prevalence between sexes, but women presented with greater hypercapnia (Palm et al., 2015). In a younger Scottish cohort (mean age ~56 years), the prevalence of OHS was similar between sexes (Arish et al., 2022). Collectively, these studies disclose an increasing risk of OHS with age in women. We found that the bicarbonate levels in women exceeded that of men after 50 years of age, but the magnitude of the difference was small, precluding firm conclusions about OHS risk.

Some studies have reported increased bicarbonate levels in OSA, in proportion to the apnea–hypopnea index (Kaw et al., 2009; Chung et al., 2013; Eskandari et al., 2017; Pei and Gui, 2021), even in the absence of OHS (Eskandari et al., 2017), in cohorts that were less likely to have OHS based on relatively low average BMIs (Chung et al., 2013; Pei and Gui, 2021) and when comparing to BMI-matched controls (Gold et al., 1993). OSA patients with frequent or long apneas may accumulate CO_2_ during sleep (Ayappa et al., 2002; Chung et al., 2013), leading to a compensatory increase of bicarbonate levels. In fact, it was recently hypothesized that higher bicarbonate levels could identify a subgroup of OSA patients with a modified chemosensory function, which could be therapeutically targeted (Hedner et al., 2022; Zou et al., 2022). Data from our cohort confirm higher bicarbonate levels in those with OSA compared to those without OSA. However, OSA status was a weaker determinant of bicarbonate levels than age, sex, or BMI. We noted that, in those with OSA and lower BMIs (i.e., in the non-obese range), the difference in bicarbonate levels between sexes was minimal. At lower BMIs, the pathophysiology of OSA is likely related to factors such as craniofacial anatomy or low arousal threshold (Eckert and Younes, 2014; Gray et al., 2017), which may overshadow any “protective” factors related to ventilation observed in women.

Our study has several imitations. First, there may be unmeasured confounders, such as comorbid conditions or medications that influence bicarbonate levels. We relied on ICD codes to exclude conditions such as renal diseases and other pulmonary disorders, which may inadequately or inaccurately capture diagnoses. Additionally, EHR data may not accurately reflect administered medications. Variations in dietary intake or blood sample handling can also affect bicarbonate results. Second, a lack of arterial blood gas data limits investigations into whether our findings reflect a primary respiratory or metabolic acid–base phenomenon. Third, patients with OSA are likely under-captured in our cohort, given the relatively low prevalence of 8% in a cohort of patients with a mean BMI of 29 kg/m^2^. Our database also lacked information about OSA severity or treatment. The rationale for capturing OSA in this study was to examine whether the subgroup of OSA patients would be more enriched for OHS and consequently higher bicarbonate levels. As such, we limited the diagnostic codes to only those that specified *obstructive* sleep apnea and did not include more generalized diagnostic codes such as sleep apnea, unspecified (ICD-10 G473.0) or other sleep apnea (ICD-10 G473.9). We acknowledge that there are inherent limitations in accurately capturing patients with OSA based on EHR data. Other recently published studies using EHR data to identify patients with OSA used similar diagnostic codes to capture OSA (Maas et al., 2021; Renno et al., 2021). Finally, while our sample size is large and is composed of patients from a national database, the generalizability of our results remains limited to patients who engaged in medical care in the participating HCOs during a prespecified period. Additionally, our analytic sample was a homogeneous population in terms of race and ethnicity. Our finding that race and ethnicity were not significant determinants of bicarbonate levels is prone to type 2 errors given the small proportion of non-white Hispanic patients. Prior studies with more diverse patients found a difference in the relationship between BMI and bicarbonate levels in Black patients compared to white patients (Lambert and Abramowitz, 2021).

## Conclusion

In summary, our cross-sectional analysis of a cohort of more than 93,000 patients revealed that serum bicarbonate levels decrease with an increase in BMI, suggesting that obesity defined by BMI is insufficient for capturing OHS risk. Additionally, we found significant sex differences in the relationship between age and serum bicarbonate levels, which may reflect the dynamic respiratory effects of progesterone and possibly estrogen over the life span of women. Menopausal status may be a risk factor indicating susceptibility to OHS. Overall, BMI is not associated with elevated bicarbonate levels, whereas age modifies the impact of sex on bicarbonate levels.

## Supplementary Material

Supplemental figures

Supplemental tabes

## Figures and Tables

**TABLE 1:** Patient characteristics stratified by sex.

Feature	Overall (*N* = 93,320)	Women (*n* = 52,569)	Men (*n* = 40,751)	*p* *
Sodium bicarbonate (mmol/L), mean (*SD*)	24.8 (2.8)	24.4 (2.8)	25.2 (2.6)	<0.001
BMI (kg/m^2^), mean (*SD*)	29.1 (6.1)	29.1 (6.4)	29.1 (5.6)	0.994
Age (years), mean (*SD*)	49.4 (17.9)	48.7 (18.1)	50.2 (17.5)	
Presence of OSA, *N* (%)	7,399 (8%)	3,105 (6%)	4,294 (11%)	<0.001
Presence of CHF, *N* (%)	6,950 (7%)	3,128 (6%)	3,822 (9%)	<0.001
**Race, *n* (%)**				<0.001
White	66,937 (72%)	37,117 (71%)	29,820 (73%)	
Black	6,565 (7%)	4,000 (8%)	2,565 (6%)	
Asian	2,128 (2%)	1,230 (2%)	898 (2%)	
American Indian	186 (0%)	110 (0%)	76 (0%)	
Missing/Other	17,504 (19%)	10,112 (19%)	7,392 (18%)	
**Ethnicity, *n* (%)**				<0.001
Hispanic	13,398 (14%)	8,239 (16%)	5,159 (13%)	
Not Hispanic	74,826 (80%)	41,636 (79%)	33,190 (81%)	

* Determined by Student’s t-test for bicarbonate levels, age, and BMI and the chi-squared test for other features.

BMI, body mass index; OSA, obstructive sleep apnea; CHF, congestive heart failure.

**TABLE 2:** Summary of the relative importance of the predictors from gradient-boosted models.

	Overall (*N* = 93,320, *R*-squared = 0.085)		Women (*n* = 52,569, *R*-squared = 0.095)		Men (*n* = 40,751, *R*-squared = 0.027)	
Feature	Fractional gain	Relative importance	Fractional gain	Relative importance	Fractional gain	Relative importance
Age	0.557	1.000	0.883	1.000	0.621	1.000
Sex (male)	0.354	0.636	-	-	-	-
BMI	0.084	0.151	0.111	0.126	0.375	0.604
OSA	0.006	0.011	0.005	0.006	0.004	0.006

When race and ethnicity were added to the models, the estimated R-squared was 0.078, 0.014, and 0.095 for the entire cohort, men, and women, respectively.

BMI, body mass index; OSA, obstructive sleep apnea.

## Data Availability

The raw data supporting the conclusions of this article will be made available by the authors, without undue reservation.
