# Supplementary material for: Effects of sex, age, and body mass index on serum bicarbonate
Source: Front Sleep. 2023 Jul 20;2:1195823. doi: 10.3389/frsle.2023.1195823 (PMC10512520; doi:10.3389/frsle.2023.1195823)
Supplement: Supplementary file 1 [file Data_Sheet_1.PDF]

Figure S1

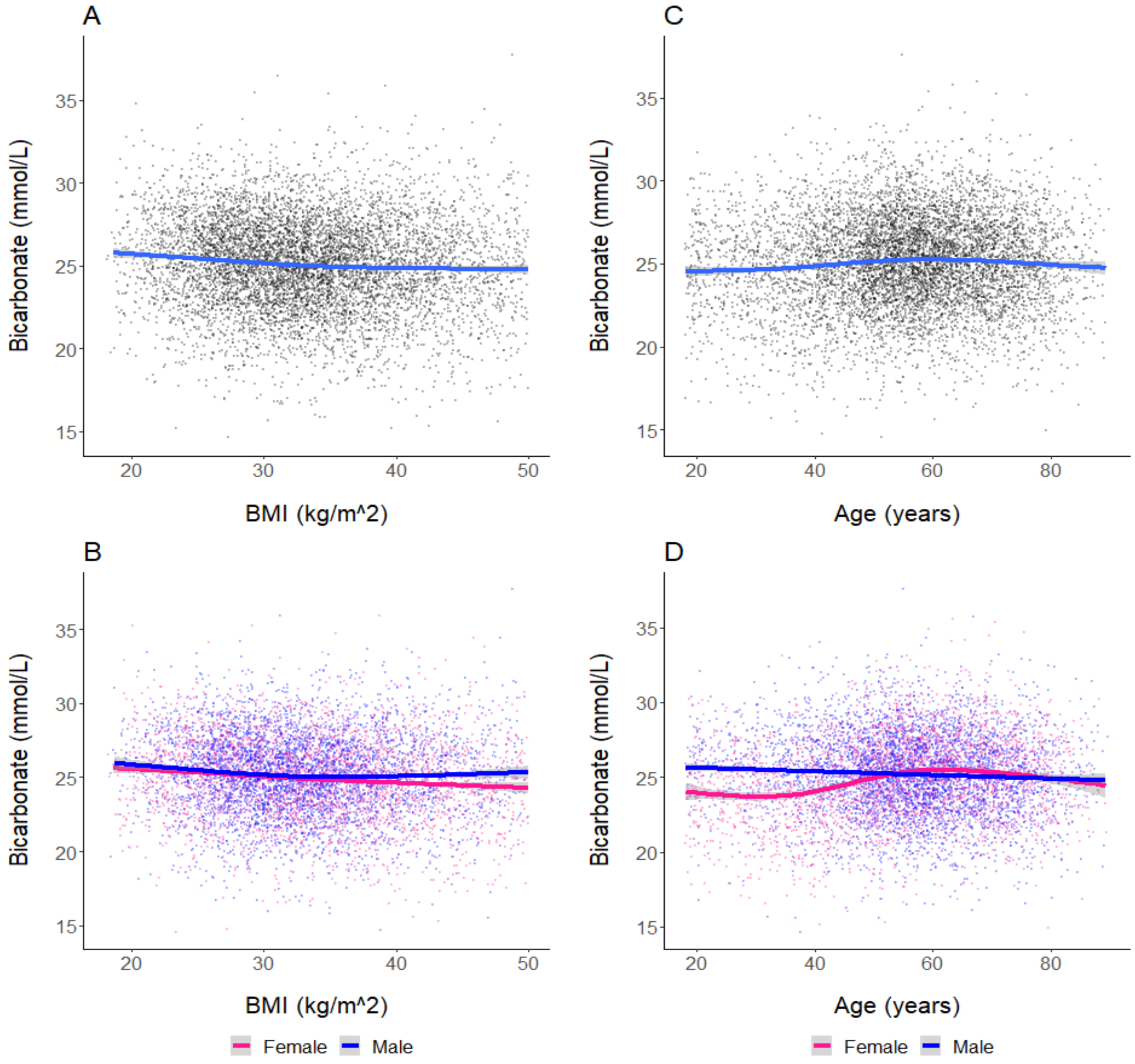

Figure S1. (A) Association between bicarbonate and BMI, only in those with OSA (n=7399). Each dot represents an individual patient measure (with 0.5 jitter applied to improve visualization of overlapping data), and the line represents a smoothed average of bicarbonate levels as a function of BMI. **(B) Association between bicarbonate and BMI stratified by sex, only in those with OSA. (C) Association between bicarbonate and age, only in those with OSA**. Each dot represents an individual patient measure (with 0.5 jitter applied to improve visualization of overlapping data), and the line represents a smoothed average of bicarbonate levels as a function of age. **(D) Association between bicarbonate and age stratified by sex, only in those with OSA**.

Figure S2

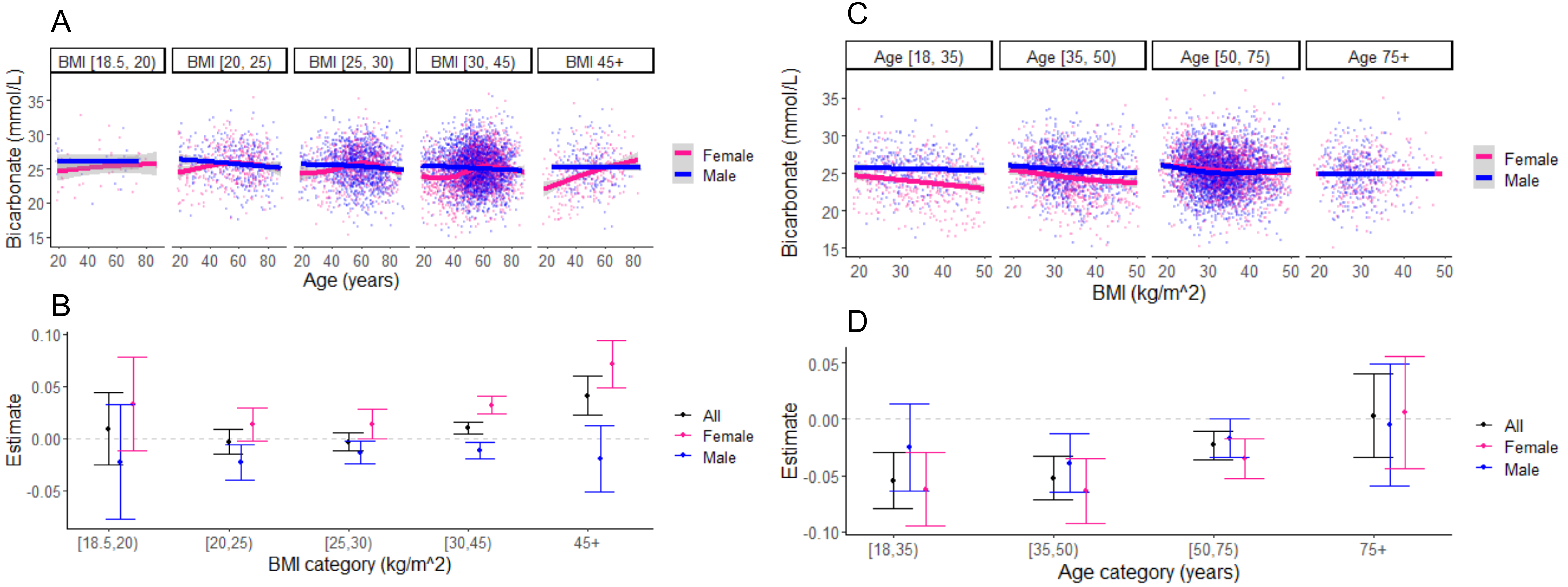

Figure S2. (A) Association between bicarbonate and age across BMI categories, stratified by sex, only in those with OSA (n=7399). Each dot represents an individual patient measure, and the line represents a smoothed average of bicarbonate levels as a function of age, within each BMI category. (B) Estimates for the effect of age on bicarbonate (outcome) from the linear regression models that include BMI categories (indicator variables), age, and the interactions between BMI categories and age. Reference BMI category was [20-25 kg/m^2). (C) **Association between bicarbonate and BMI across age categories, stratified by sex, only in those with OSA.** Each dot represents an individual patient measure, and the line represents a smoothed average of bicarbonate levels as a function of BMI, within each age category. (D) Estimates for the effect of BMI on bicarbonate (outcome) from the linear regression models that include age categories (indicator variables), BMI, and the interactions between age categories and BMI. Reference age category was [18-35 years). [indicates inclusive; (indicates exclusive.

Figure S3

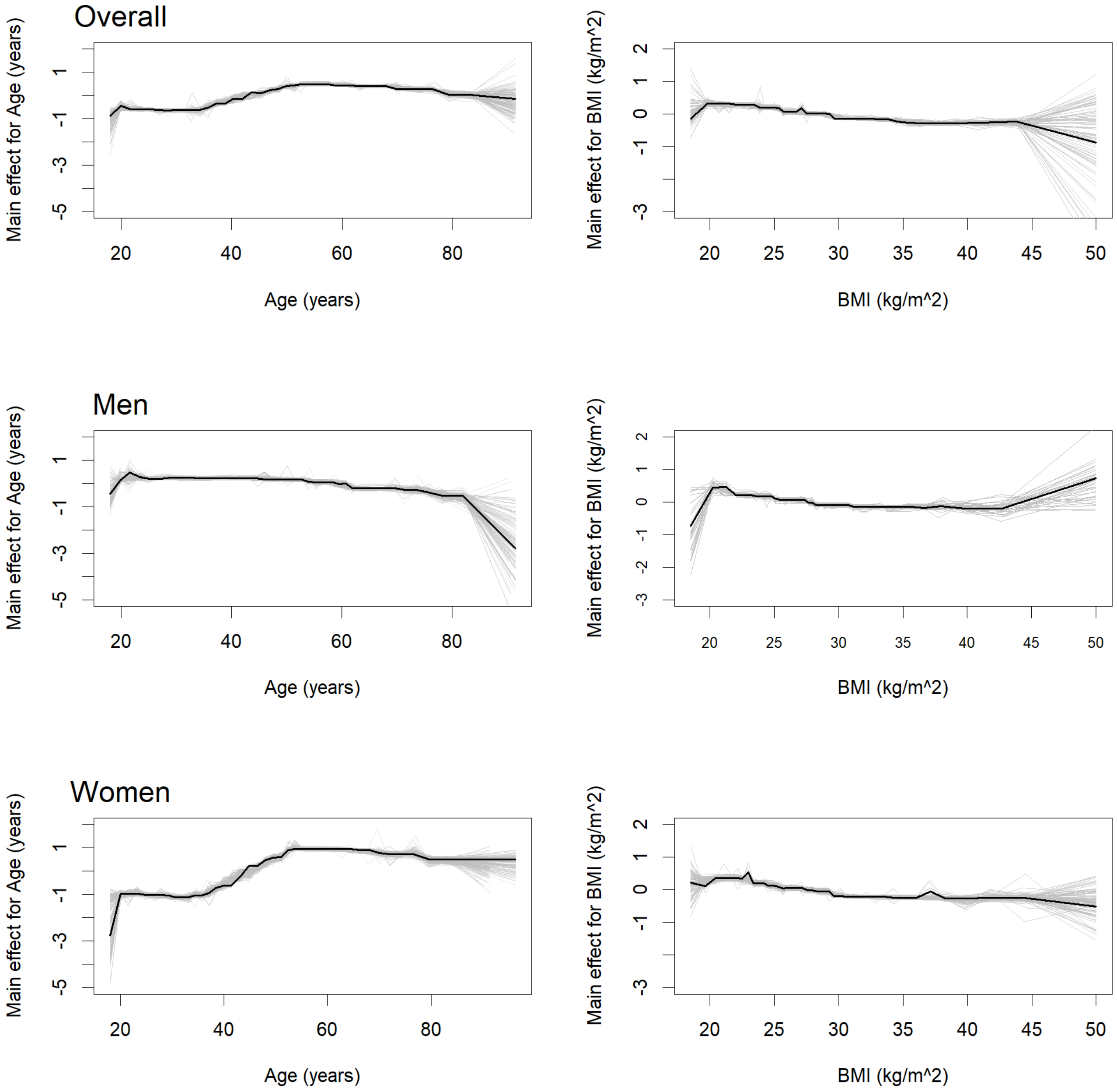

Figure S3. Accumulated local effect of age in years and BMI among 93,320 patients with measured bicarbonate. Average effect is shown in bold, Montecarlo estimated effect is shown in grey.

Figure S4

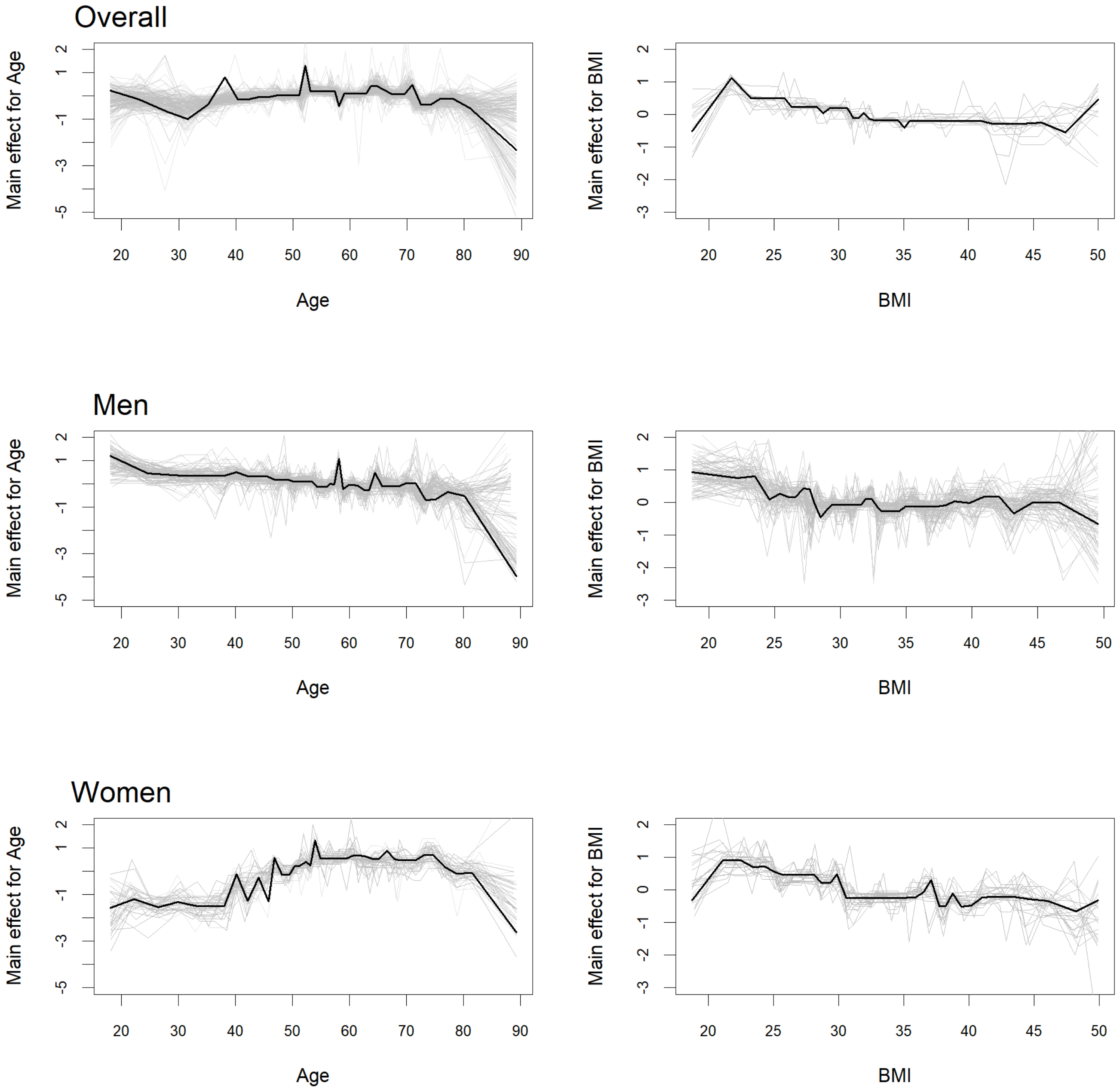

Figure S4. Accumulated local effect of age in years and BMI only in OSA subgroup (n=7399). Average effect is shown in bold, Montecarlo estimated effect is shown in grey.

Figure S5

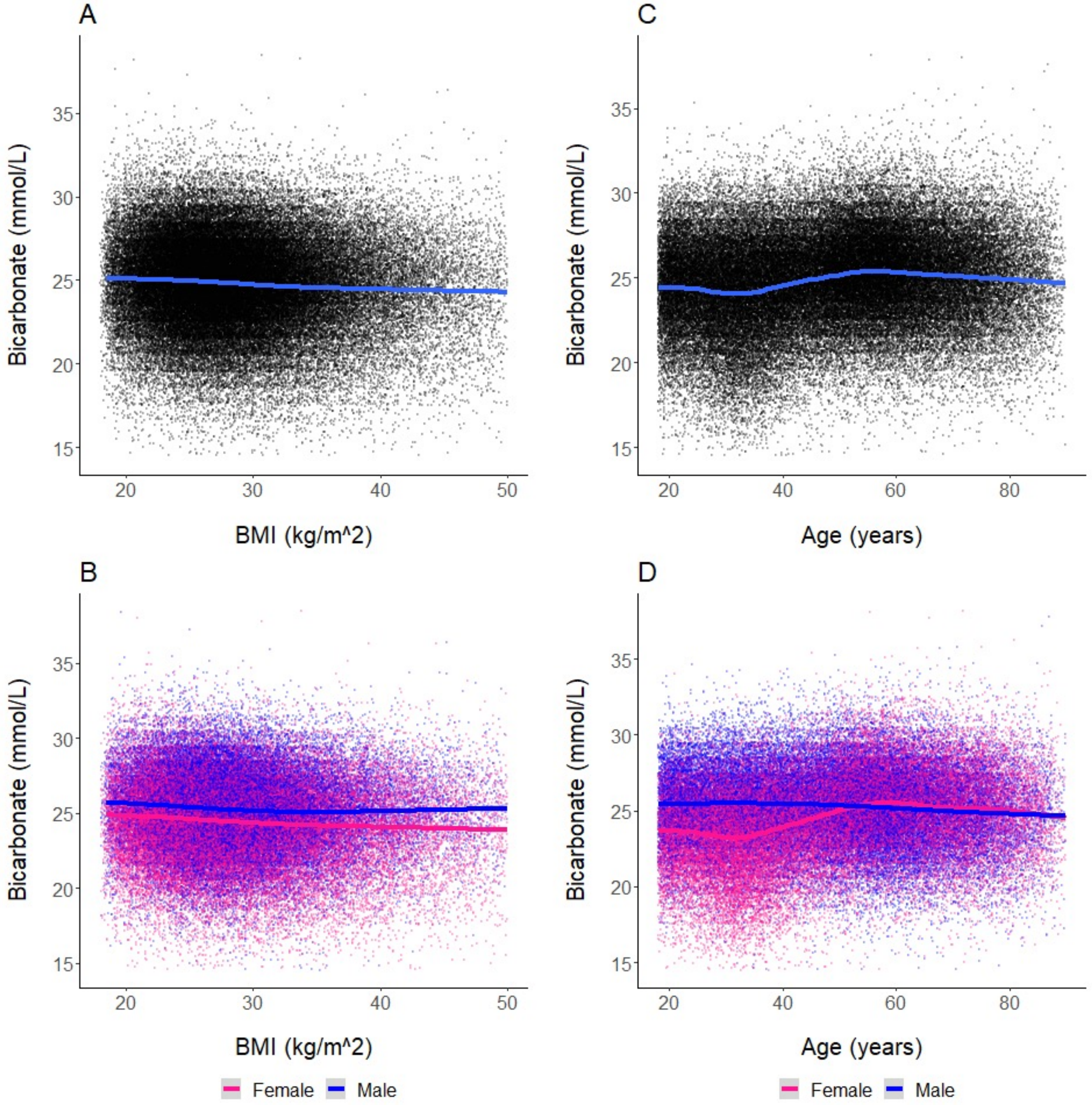

Figure S5. (A) Association between bicarbonate and BMI, only in those without CHF (n=86,370). Each dot represents an individual patient measure (with 0.5 jitter applied to improve visualization of overlapping data), and the line represents a smoothed average of bicarbonate levels as a function of BMI. **(B) Association between bicarbonate and BMI stratified by sex, only in those without CHF**. **(C) Association between bicarbonate and age, only in those without CHF**. Each dot represents an individual patient measure (with 0.5 jitter applied to improve visualization of overlapping data), and the line represents a smoothed average of bicarbonate levels as a function of age. **(D) Association between bicarbonate and age stratified by sex, only in those without CHF**.
