# Supplementary material for: Effects of sex, age, and body mass index on serum bicarbonate
Source: Front Sleep. 2023 Jul 20;2:1195823. doi: 10.3389/frsle.2023.1195823 (PMC10512520; doi:10.3389/frsle.2023.1195823)
Supplement: Supplementary file 2 [file Table_1.DOCX]

| **Table S1. Linear regression estimates for the association between bicarbonate and age across BMI categories** | | | | |
| --- | --- | --- | --- | --- |
|  |  |  |  |  |
|  | **N** | **Estimate** | **95% Confidence Interval** | **Pr(>\|t\|)** |
| **Overall (R-squared = 0.036)** | | |  |  |
| (Intercept) |  | 24.25 | (24.16, 24.34) | <0.001 |
| bmi.catlt20 |  | -0.31 | (-0.44, -0.18) | <0.001 |
| bmi.cat[25,30) |  | -1.32 | (-1.45, -1.19) | <0.001 |
| bmi.cat[30,45) |  | -2.44 | (-2.86, -2.01) | <0.001 |
| bmi.cat45+ |  | -0.20 | (-0.46, 0.05) | 0.120 |
| male |  | 0.73 | (0.70, 0.77) | <0.001 |
| Age* | 23078 | 0.01 | (0.01, 0.01) | <0.001 |
| bmi.catlt20:Age | 2695 | 0.01 | (0.00, 0.01) | 0.011 |
| bmi.cat[25,30):Age | 31882 | 0.00 | (-0.00, 0.00) | 0.944 |
| bmi.cat[30,45):Age | 34019 | 0.02 | (0.01, 0.02) | <0.001 |
| bmi.cat45+:Age | 1646 | 0.04 | (0.03, 0.05) | <0.001 |
| **Among Men (R-squared = 0.013)** | | | |  |
| (Intercept) |  | 26.17 | (26.03, 26.30) | <0.001 |
| bmi.catlt20 |  | -0.14 | (-0.32, 0.05) | 0.163 |
| bmi.cat[25,30) |  | -0.65 | (-0.85, -0.45) | <0.001 |
| bmi.cat[30,45) |  | -1.08 | (-1.89, -0.27) | 0.009 |
| bmi.cat45+ |  | -0.40 | (-0.80, -0.00) | 0.048 |
| Age* | 8881 | -0.01 | (-0.02, -0.01) | <0.001 |
| bmi.catlt20:Age | 883 | 0.01 | (-0.00, 0.02) | 0.066 |
| bmi.cat[25,30):Age | 15591 | 0.00 | (-0.01, 0.00) | 0.194 |
| bmi.cat[30,45):Age | 14860 | 0.01 | (0.00, 0.01) | 0.010 |
| bmi.cat45+:Age | 536 | 0.02 | (0.00, 0.04) | 0.027 |
| **Among Women (R-squared = 0.066)** | | | |  |
| (Intercept) |  | 23.43 | (23.31, 23.55) | <0.001 |
| bmi.catlt20 |  | -0.63 | (-0.80, -0.46) | <0.001 |
| bmi.cat[25,30) |  | -1.61 | (-1.77, -1.44) | <0.001 |
| bmi.cat[30,45) |  | -2.49 | (-2.98, -1.99) | <0.001 |
| bmi.cat45+ |  | 0.04 | (-0.28, 0.37) | 0.796 |
| Age* | 14197 | 0.03 | (0.02, 0.03) | <0.001 |
| bmi.catlt20:Age | 1812 | 0.00 | (-0.00, 0.01) | 0.608 |
| bmi.cat[25,30):Age | 16291 | 0.01 | (0.00, 0.01) | <0.001 |
| bmi.cat[30,45):Age | 19159 | 0.02 | (0.02, 0.03) | <0.001 |
| bmi.cat45+:Age | 1110 | 0.04 | (0.03, 0.05) | <0.001 |
| * for reference BMI category, 20-25;  [ indicates inclusive; ( indicates exclusive. | | | | |

| **Table S2. Linear regression estimates for the association between bicarbonate and BMI across age categories** | | | | |
| --- | --- | --- | --- | --- |
|  |  |  |  |  |
|  | **N** | **Estimate** | **95% Confidence Interval** | **Pr(>\|t\|)** |
| **Overall (R-squared = 0.039)** |  |  |  |  |
| (Intercept) |  | 25.72 | (25.57, 25.87) | <0.001 |
| age.cat[35,50) |  | -0.25 | (-0.48, -0.02) | 0.030 |
| age.cat[50,75) |  | -0.36 | (-0.56, -0.15) | 0.001 |
| age.cat75+ |  | -1.41 | (-1.76, -1.05) | <0.001 |
| Male |  | 0.68 | (0.65, 0.72) | <0.001 |
| BMI* | 24609 | -0.06 | (-0.07, -0.06) | <0.001 |
| age.cat[35,50):BMI | 22425 | 0.03 | (0.02, 0.03) | <0.001 |
| age.cat[50,75):BMI | 38063 | 0.04 | (0.04, 0.05) | <0.001 |
| age.cat75+:BMI | 8223 | 0.07 | (0.06, 0.08) | <0.001 |
| **Among Men (R-squared = 0.012)** |  |  |  |  |
| (Intercept) |  | 26.56 | (26.31, 26.81) | <0.001 |
| age.cat[35,50) |  | -0.25 | (-0.62, 0.13) | 0.195 |
| age.cat[50,75) |  | -1.30 | (-1.63, -0.97) | <0.001 |
| age.cat75+ |  | -1.26 | (-1.86, -0.65) | <0.001 |
| BMI* | 9689 | -0.04 | (-0.05, -0.03) | <0.001 |
| age.cat[35,50):BMI | 9518 | 0.01 | (-0.00, 0.02) | 0.143 |
| age.cat[50,75):BMI | 18213 | 0.03 | (0.02, 0.04) | <0.001 |
| age.cat75+:BMI | 3331 | 0.02 | (-0.01, 0.04) | 0.162 |
| **Among Women (R-squared = 0.077)** | | | |  |
| (Intercept) |  | 25.17 | (24.99, 25.36) | <0.001 |
| age.cat[35,50) |  | 0.22 | (-0.07, 0.50) | 0.137 |
| age.cat[50,75) |  | 0.71 | (0.45, 0.97) | <0.001 |
| age.cat75+ |  | -0.83 | (-1.26, -0.39) | <0.001 |
| BMI* | 14920 | -0.06 | (-0.07, -0.05) | <0.001 |
| age.cat[35,50):BMI | 12907 | 0.02 | (0.01, 0.03) | <0.001 |
| age.cat[50,75):BMI | 19850 | 0.04 | (0.03, 0.05) | <0.001 |
| age.cat75+:BMI | 4892 | 0.08 | (0.06, 0.10) | <0.001 |
| * for reference age 18 to 35 years   [ indicates inclusive; ( indicates exclusive. | | | | |

| **Table S3. Patient characteristics stratified by obstructive sleep apnea status.** | | | | |
| --- | --- | --- | --- | --- |
|  |  |  |  |  |
| **Feature** | **No OSA (n=85921)** | **OSA (n=7399)** | **p*** |  |
| Sodium bicarbonate (mmol/L); mean (SD) | 24.7 (2.8) | 25.1 (2.7) | <0.001 |  |
| BMI (kg/m^2^); mean (SD) | 28.7 (5.9) | 33.3 (6.7) | <0.001 |  |
| Age (years); mean (SD) | 48.8 (18) | 55.6 (14.5) | <0.001 |  |
| Male; N (%) | 36457 (42%) | 4294 (58%) | <0.001 |  |
| **Race; N (%)** |  |  | <0.001 |  |
| White | 61010 (71%) | 5927 (80%) |  |  |
| Black | 6129 (7%) | 436 (6%) |  |  |
| Asian | 2029 (2%) | 99 (1%) |  |  |
| American Indian | 171 (0%) | 15 (0%) |  |  |
| Missing/Other | 16582 (19%) | 922 (12%) |  |  |
| **Ethnicity; N (%)** |  |  | <0.001 |  |
| Hispanic | 12753 (15%) | 645 (9%) |  |  |
| Not Hispanic | 68326 (80%) | 6500 (88%) |  |  |
| * Determined by Student's t test for bicarbonate, age, and BMI, and Chi square test for other features.  BMI, body mass index; OSA, obstructive sleep apnea | | | |  |
|  |  |  |  |  |

| **Table S4. Patient characteristics stratified by congestive heart failure status.** | | | | |
| --- | --- | --- | --- | --- |
| **Feature** | | **No CHF (n=86370)** | **CHF (n=6950)** | **p*** |
| Sodium bicarbonate (mmol/L); mean (SD) | | 24.8 (2.7) | 24.4 (2.8) | <0.001 |
| BMI (kg/m^2^); mean (SD) | | 29 (6.1) | 29.7 (6.1) | <0.001 |
| Age (years); mean (SD) | | 48.1 (17.4) | 65.7 (14.6) | <0.001 |
| Male; N (%) | | 36929 (43%) | 3822 (55%) | <0.001 |
| **Race; N (%)** | |  |  | <0.001 |
| White | | 61312 (71%) | 5625 (81%) |  |
| Black | | 6175 (7%) | 390 (6%) |  |
| Asian | | 2009 (2%) | 119 (2%) |  |
| American Indian | | 174 (0%) | 12 (0%) |  |
| Missing/Other | | 16700 (19%) | 804 (12%) |  |
| **Ethnicity; N (%)** | |  |  | <0.001 |
| Hispanic | | 12890 (15%) | 508 (7%) |  |
| Not Hispanic | | 68697 (80%) | 6129 (88%) |  |
|  |  |  |  |  |
| CHF, congestive heart failure | |  |  |  |
